# Editorial: Bioinspired Functional Surfaces with Superwettability: From Fabrication to Applications

**DOI:** 10.3389/fchem.2021.658572

**Published:** 2021-03-23

**Authors:** Jiale Yong

**Affiliations:** School of Electronic Science and Engineering, Xi’an Jiaotong University, Xi’an, China

**Keywords:** superwettability, superhydrophilicity, superhydrophobicity, superoleophobicity, superaerophobicity

**Editorial on the Research Topic**

**Bioinspired Functional Surfaces with Superwettability: From Fabrication to Applications**

Surface wettability is a basic property of solid materials, which is mainly determined by both the surface chemical composition and physical morphology. Recently, the materials with superwettability have attracted increasing attention because of their rich practical applications, such as liquid resistance, self-cleaning, anti-icing, droplet manipulation, oil/water separation, fog harvest, microfluidics, gas collection, drag reduction, and cell engineering. Many animals and plants have evolved special surface wettability. Inspired by nature, various artificial surfaces with extreme wettabilities (such as superhydrophilicity, superhydrophobicity, superoleophilicity, superoleophobicity, superaerophilicity, superaerophobicity, and slippery property) have been developed until now. This Research Topic gives a collection of reviews and research articles on the fabrication and applications of the superwetting surfaces.

This special issue contains five reviews and two original research articles. Yong et al. discuss and clarify the relationship between six kinds of superwettabilities to water, oil, and gas, respectively. Usually, a superhydrophilic surface also shows superoleophobic and superaerophobic properties in water. By contrast, a superhydrophobic surface also shows superoleophilic and superaerophilic properties underwater. The superwettability of a textured surface can convert between superhydrophobicity and superhydrophilicity by simple hydrophilic and hydrophobic treatment. Therefore, we can realize various superwettabilities (for example, superhydrophilic, superhydrophobic, underwater superoleophilic, underwater superoleophobic, underwater superaerophilic, and underwater superaerophobic properties) on a rough microstructure and reversibly switch surface wettability from one state to other states.

Some smart materials can respond to external stimulation, which have been explored to achieve switchable wettability. Han et al. summarize the recent progress of the switchable wettability based on the stimulus-responsive materials. The wettability of those smart surfaces can reversibly switch through changing the surface chemistry or surface roughness upon external stimulation (such as thermal, light, electrical field, magnetic field, and mechanical motion).

Liu et al. briefly introduce three laser patterning methods to achieve superhydrophobic surfaces. The laser can process various materials and make geometrical modifications on those substrates. As a result, superhydrophobicity is easily obtained on laser-processed surfaces. Graphene is growing to a star material in the past decade. Ma et al. review the recent advances in the fabrication of superwetting graphene surfaces by laser processing. The multi-functions of the superwetting graphene surfaces are also introduced.

Regarding the application examples, the integration of superwetting micro or nanostructures can endow optical devices with anti-liquid and self-cleaning properties. Bian et al. prepare an underwater superoleophobic microlens array on an optical glass. The resultant oil-repellent microlens array has a self-cleaning capacity and still keeps great imaging ability in the water. Yong et al. further summarize the strategies of producing superwetting optical devices (e.g., microlens array and artificial compound eye) and show how to integrate an optical device with the additional superhydrophobicity and superoleophobicity. One is to design a hybrid patterned structure which is consisted of both the optical-element domain and the liquid-repellent domain. Another is to directly generate superwetting fine structures on the surface of the optical devices.

As another example, Yong et al., 2020 separate the mixture of water and oil by using a superhydrophilic and underwater superoleophobic porous membrane. When the oil/water mixture is poured onto the superwetting porous membrane, the superhydrophilicity allows the water to quickly penetrate the membrane, whereas the oil in the mixture is intercepted by the prewetted membrane because of the underwater superoleophobicity. So, the mixture of water and oil is successfully separated in such a high-efficiency manner.

Superwetting surfaces have a wide range of applications. The contributions in this special issue focus on the fabrication and applications of various kinds of superwetting surfaces. The design, fabrication, and broad applications of superwetting materials are still the major focus in this research field. We believe that an exciting future in the design, realization, and application of more complicated and subtle superwettability will be witnessed, because of their great commercialization value and potential.
